# Molecular Mechanisms of Gene Expression Regulation in Response to Heat Stress in *Hemerocallis fulva*

**DOI:** 10.3390/plants14050690

**Published:** 2025-02-24

**Authors:** Boyan Chu, Weixue Liu, Jinxia Li, Xiaofei Zhang, Ping Li

**Affiliations:** 1 Hebei Academy of Forestry and Grassland Science, Shijiazhuang 050061, China; liuweixue2023@163.com (W.L.); lijinxia299@163.com (J.L.); zxf202310@126.com (X.Z.); 2 Hebei Key Laboratory of Floral Biological Breeding, Hebei Agricultural University, Baoding 071000, China; 3 College of Landscape and Tourism, Hebei Agricultural University, Baoding 071000, China; 4 College of Forestry, Hebei Agricultural University, Baoding 071000, China

**Keywords:** *Hemerocallis fulva*, heat stress, transcriptome, WGCNA(Weighted Gene Co-expression Network Analysis) analysis, molecular mechanism

## Abstract

*Hemerocallis fulva* is one of the three major flowers in the world; its flower type and color are very rich, with high ornamental value and economic value. Heat stress severely limits the cultivation and geographical distribution of *H. fulva*. Genetic resources and their underlying molecular mechanisms constitute the cornerstone of contemporary breeding technologies. However, research on the response of *H. fulva* to heat stress remains relatively scant. In this study, we used the heat-resistant ‘Dan Yang’ variety and heat-sensitive ‘Nuo Mi Lu’ variety with phenotypic expression as experimental materials to determine the changes in substance and gene expression levels, and used bioinformatics technology to study the molecular mechanisms and gene resource mining of *H. fulva* in response to heat stress. We identified several thousand differentially expressed genes (DEGs) in different comparison groups. At the same time, 1850 shared DEGs were identified in two *H. fulva* genotypes responding to heat stress. The dynamic cutting algorithm was used to cluster the genes, and 23 gene co-expression modules were obtained. The MEorangered, MElightpink, and MEmagenta modules were significantly correlated with physiological and biochemical traits. We identified ten key genes closely related to the response of *H. fulva* to heat stress, including plant–pathogen interactions, plant hormone signal transduction, oxidative transduction phosphorylation, and the plant hormone signal transduction pathway. This study not only analyzes the molecular mechanism of *H. fulva* response to heat stress, but also provides genetic resources for breeding *H. fulva* heat tolerance.

## 1. Introduction

High temperature is one of the main factors that affect the growth rate of plants and also one of the main abiotic factors that hinder the growth and development of plants and limit the yield and geographical distribution of crops [1,2]. With global warming, the frequency of extreme heat events has had a significant impact on plant growth and yield [3]. Heat stress refers to the phenomenon that when the ambient temperature exceeds the optimal temperature threshold for plant growth and lasts for a period of time, the physiological function of the plant is irreversibly damaged [4,5]. As an important adverse environmental stress, heat stress not only affects the growth and development of *H. fulva*, but also has significant effects on its external morphology and internal physiological structure [6]. When the ambient temperature rises to a certain degree, *H. fulva* may suffer irreversible damage. For example, high temperatures have a great impact on photosynthesis [7], resulting in an increase in the reactive oxygen species (ROS) content of the chlorophyll inside the plant and causing photoinhibition [8]. High temperatures can also affect the time of day and duration of flowering, and raising the temperature usually encourages the flowers to open [9].

The change in plant gene expression level under heat stress is a complex network system involving multiple gene expression regulation [10,11]. Transcriptome studies can help us systematically understand the dynamic changes in gene expression in plants under heat stress. At present, transcriptome sequencing has been widely used to study the molecular mechanisms of plant heat stress in many plants, such as *Persea americana* [12], *Brassica rapa* [13], *Populus euphratica* [14], wheat [15], etc. The massive synthesis of heat shock proteins (HSPs) was found in the leaves, stems, and roots of potato using transcriptome sequencing [16]. In addition, it was found that small heat shock proteins (sHSPs) in plants are not expressed at room temperature, but are rapidly synthesized under heat shock conditions, which plays an important role in maintaining the stability of plant cell proteins and biofilm structure [17]. Transcriptome sequencing has revealed multiple genes and metabolic pathways associated with plant heat stress, and these findings provide an important molecular basis for understanding how plants adapt to high temperatures and provide potential genetic resources for breeding heat-resistant plants.

At present, the response of plants to high-temperature stress has been studied deeply. A regulatory network for the response to heat stress in Arabidopsis thaliana has been established [18]. After research, heat shock factor and heat shock protein were found to activate heat shock response (*HSR*), and some heat reaction signaling molecules (such as calcium ion Ca^2+^, nitric oxide NO, and hormones) that affect the upstream of the heat shock response play a key role [19,20]. *HSR* gene expression is also regulated by non-coding RNA (ncRNA) and epigenetic regulation [18]. The endoplasmic-reticulum-unfolded protein response plays an important role in heat stress response [21]. Mitochondria and chloroplasts, as heat-sensitive organelles, respond to heat stress through a variety of mechanisms [22]. In the process of growth and development, *H. fulva* have also developed a variety of conservative and effective defense measures to deal with high temperatures to protect themselves from injury. For example, *H. fulva* can promote the synthesis of heat shock proteins [18], downregulate proteasome activity, and regulate metabolic patterns in high-temperature environments. There are also 11 endophytes in the *H. fulva* that help the plant develop and tolerate different abiotic stresses by secreting a variety of compounds that promote plant growth, such as auxin, gibberellin, ferrite, and cytokinin [23,24].

*H. fulva* is native to southern China, but is also found in Siberia, Japan, and Southeast Asia. H. fulva is one of the three major flowers in the world; its flower type and color are very rich [25], with high ornamental value [26] and economic value [27]. It is widely used in landscaping and home decoration [28]. Fresh-cut and potted *H. fulva* produce significant economic value every year. And *H. fulva* can be used to treat diseases [29]. It has a very long history of application as a vegetable and even as a medicinal plant, having a history of thousands of years [30]. However, there are few studies on the discovery and utilization of genes related to the heat resistance of *H. fulva*. In this study, two varieties of “Dan Yang” and “Nuo Mi Lu” were selected for heat stress treatment using the determination of morphological and physiological indexes. Transcriptomic sequencing was used to identify DEGs related to heat stress in *H. fulva*, related genes were screened based on WGCNA, and the functions of the selected key genes were verified using qPCR techniques in order to provide theoretical basis and data support for the development and utilization of resources and breeding of stress-resistant varieties of *H. fulva*.

## 2. Materials and Methods

### 2.1. Plant Materials and Heat Treatments

The new breed ‘Dan Yang’ (DY), with the ascension number JI S-SV-HF-038-2024, and the introduced variety ‘Nuo Mi Lu’ (NML) of *H. fulva* were selected by Hebei Academy of Forestry and Grassland Science as experimental materials. When the material grew to the 6th week and the number of leaves per plant was 8–10, it was randomly placed into two artificial climate chambers for normal maintenance for 1 week. The climate chamber was set at 25 °C, 16 h/8 h (day/night), relative humidity was 65%, light was 200 μmol m^−1^·s^−1^, and normal water management was maintained. In this experiment, the two varieties were treated at 40 °C for 0 h (CK control group), 4 h, 8 h, 12 h, 24 h, 48 h, and 72 h, and each group was repeated three times.

### 2.2. Determination of Physiological Indexes of H. fulva Under Heat Stress

The superoxide dismutase (SOD) assay utilized the WST-8 method, which could effectively react with the superoxide anion (O_2_^−^) generated by catalyzing xanthine oxidase (XO) to produce a water-soluble formazan dye [31]. This method was now more stable and sensitive. Peroxidase (POD) was a ubiquitous and important oxidoreductase, and its activity was closely related to the resistance. POD activity was determined using the Guaiacol method, while the catalase (CAT) activity was measured using the UV spectrophotometric method [32]. Sugars under the action of concentrated sulfuric acid by dehydration reaction to generate furfural or hydroxymethylfurfural, generated by furfural or hydroxymethylfurfural and then dehydrated with anthrone condensation. The formation of a blue-green material in the visible region of the maximum absorption of the furfural derivatives at a wavelength of 620 nm, and its light absorption value in a certain range and the content of sugar was proportional to the relationship. The sugar content measured using this method was the total soluble sugar content. Soluble protein content was measured by the Coomassie brilliant Blue method, malondialdehyde (MDA) content were determined using the thiobarbituric acid method, and the levels of free proline were measured using the colorimetric measurement method [33].

### 2.3. Transcriptome Sequencing

In order to study the molecular mechanism of heat stress in *H. fulva*, ‘Danyang’ and ‘Nuoelu’ were treated under 25 °C control conditions (CK), 40 °C heat stress conditions for 12 h and 48 h, and the leaves of two plants (CK1, CK2) of two varieties were taken under 25 °C conditions. Three biological replicates were performed in each control group and treatment group. A total of 24 samples were sequenced using transcriptome. The total RNA was extracted from the experimental materials using an RNA extraction kit. The cDNA library of *H. fulva* was constructed using the method of Oligo (dT) magnetic bead enrichment. The quality of the library was detected using a Qubit 3.0 fluorescence quantitative analyzer, Qsep400 high-throughput analysis system, and the Q-PCR method, respectively. After the library passed the quality inspection, the transcriptome of *H. fulva* was sequenced using the PE150 mode of the Illumina NovaSeq6000 sequencing platform.

### 2.4. Transcriptome Data Analysis

Using a Perl script, the raw data in FASTQ format (raw reads) was processed. Clean data were obtained by removing reads containing adapters, reads with poly-N sequences, and low-quality reads from the raw data. Additionally, the Q20, Q30, GC content, and sequence duplication levels of the clean data were calculated. The RNA-seq sequencing data were assembled using the Trinity (2.14.0) software [34]. The assembly results were then optimized and quantified using the RSEM (v1.2.19) software [35]. The completeness of the assembly was assessed using Benchmarking Universal Single-Copy Orthologs (BUSCO) [36]. The quality of the assembly results was evaluated based on the N50 value. A longer Unigene N50 indicated better assembly quality. In this study, a total of 48,511 Unigenes were obtained, with an N50 of 2483, suggesting a high level of assembly completeness.

The Unigene sequences were aligned against the NR [37], Swiss-Prot [38], COG [39], KOG [40], eggNOG4.5 [41], and KEGG [42] databases using the DIAMOND (v2.0.4) [43] software. The KEGG Orthology results of the Unigenes in the KEGG database were obtained using KOBAS [44]. The GO Orthology results of the new genes were analyzed using InterProScan with the integrated databases from InterPro [45]. After predicting the amino acid sequences of the Unigenes, they were aligned against the Pfam database [46] using the HMMER (v3.1b2) software [47] to obtain the annotation information of the Unigenes.

### 2.5. H. fulva Heat-Tolerance-Related Gene Analysis

Differential expression analysis was performed using the EBSeq (v1.30.0) software [48]. DEGs were screened based on |log_2_(fold change)| ≥ 2 and FDR (false discovery rate) < 0.01, and all DEGs in ‘Dan Yang’ and ‘Nuo Mi Lu’ were retrieved. The specific and shared DEGs between the two varieties were identified using the jvenn website (https://jvenn.toulouse.inrae.fr/app/example.html (accessed on 18 October 2024)). Gene expression patterns were analyzed and visualized using the OmicShare BioCloud platform (https://www.omicshare.com/tools/Home/Task (accessed on 20 October 2024)), and the correlation of gene expression levels was analyzed using the ggplot2 package. We conducted a gene expression trend analysis using the dynamic trend analysis tool on the OmicShare platform (https://www.omicshare.com/tools/Home/Soft/getsoft (accessed on 24 October 2024)), with the dynamic trend analysis tool parameters set to detect gene expression changes with a minimum fold change in two and a *p*-value less than 0.05.

### 2.6. WGCNA Analysis

Further explore the regulatory network between genes through WGCNA [49] analysis, aiming to identify relevant gene modules and hub genes within the modules. After filtering genes with FPKM < 1, a total of 29,903 genes were used to construct the weighted gene co-expression network. When the optimal soft threshold β = 30, R^2^ > 0.80, and the average connectivity approaches 0, indicating that this soft threshold was suitable for constructing the scale-free network (Figure S1). Set the parameters to weighted network = unsigned, hierarchal clustering tree = dynamic hybrid tree cut algorithm, power = 25, minimum module size = 30, minimum height for merging modules = 0.25. Evaluate each gene module, test the correlation of genes within each module, visualize the results using Cytoscape (v3.9.1), and calculate and filter the top 150 core genes based on the MCC algorithm in the cytoHubba plugin. Calculate the characteristic gene values for each module and test the correlation between the heat tolerance of each gene. In addition, to further explore the functions of genes within the modules, the KOBAS3.0 software was used for KEGG enrichment analysis [44].

### 2.7. Real-Time Fluorescence Quantitative PCR (qRT-PCR) Analysis

In order to verify the detection of DEGs using RNA-seq sequencing, 10 DEGs were selected for qRT-PCR analysis based on analysis. Primers were designed using the online website of Primer3plus (https://www.primer3plus.com/index.html (accessed on 8 November 2024)) and were synthesized by Shanghai Shenggong Biological Company. The specific information of primers is shown in Table 1. The RNA was then reverse-transcribed into cDNA using the Fasting King RT Kit (With gDNA) reverse transcription kit (Tiangen). There were 3 replicates for each sample. qRT-PCR analysis was performed using the FastStart Essential DNA Green Master kit according to its instructions. The reaction procedure was 94 °C for 30 s, 94 °C 5 s, 60 °C 30 s, 40 cycles. The stability of *Actin* was evaluated using Genorm [50]. Using *Actin* as a reference gene, the relative expression was calculated using the 2^−ΔΔCT^ formula.

## 3. Results

### 3.1. The Effect of Heat Stress on Physiological Indices of H. fulva

**Figure 1:**
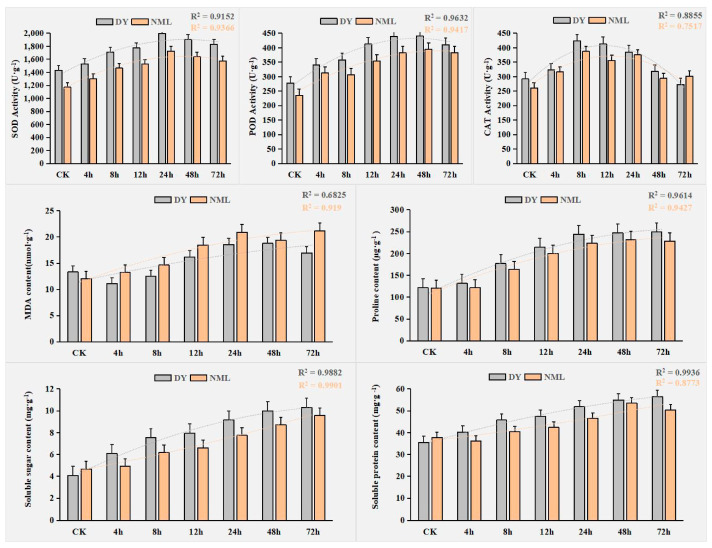
Changes in physiological indicators of *H. fulva* at different stages under heat stress (40 °C). CK represents the control treatment, where plants were not subjected to heat stress (40 °C); DY represents ‘Dan Yang’; NML represents ‘Nuo Mi Lu’. SOD stands for Superoxide Dismutase, POD stands for Peroxidase, CAT stands for Catalase, and MDA stands for Malondialdehyde. The abscissa represents time, which is 4 h, 8 h, 12 h, 24 h, 48 h, and 72 h, respectively. The ordinate represents the activity or content of each physiological index (unit: as shown in the figure). The R^2^ value in the figure represents the coefficient of determination of data fitting, reflecting the degree of data fitting.

The activity of SOD, POD, and CAT and the content of MDA, proline, soluble sugar, and soluble protein were determined, and the changes in the internal substances of *H. fulva* were further studied under heat stress. The results show that the SOD and POD activities increased with increasing time under heat stress treatment, while the SOD activity reaching its maximum value at 24 h, followed by a gentle decline. POD reached its maximum value at 48 h and then declined. The CAT activity increased rapidly under heat stress and showed a rapid decline after a plateau after 8 h. MDA, proline, soluble sugar, and soluble protein contents generally consistently increased with time (Figure 1). It was inferred that, during the response process to heat stress, there were significant changes in the substances within daylilies to cope with adverse growth conditions. At the same time, the physiological changes induced by heat stress were closely related to the duration of the stress.

### 3.2. H. fulva Heat Stress Transcriptome Sequencing

A total of 24 samples were sequenced from the transcriptome, resulting in 181.35 Gb of clean data. The clean data for each sample reached 6.49 Gb, and the percentage of Q30 bases was 91.80% or higher (Table S1).

**Figure 2:**
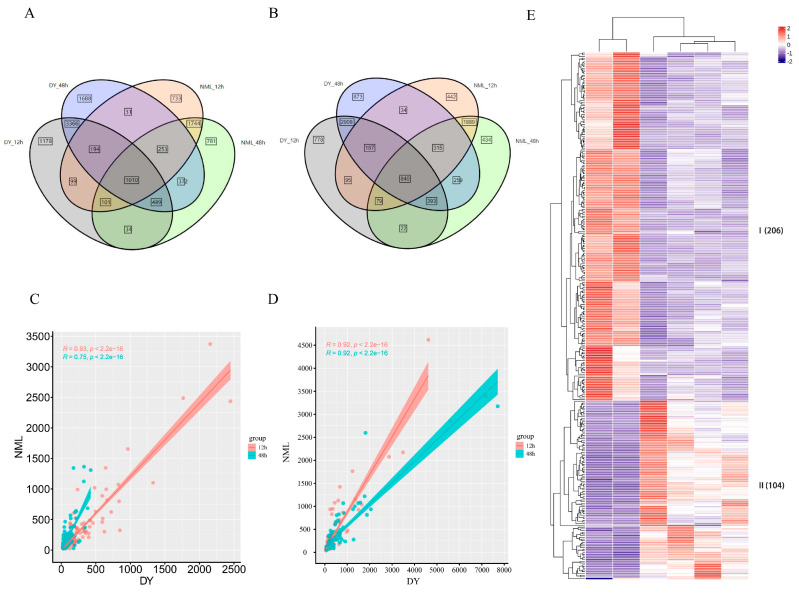
The differential expression genes in the transcriptome of Hemerocallis under heat stress. (**A**) Venn diagram representing the shared and unique upregulated genes in the heat stress response of *H. fulva* DEGs. (**B**) Venn diagram representing the shared and unique downregulated genes in the heat stress response of *H. fulva* DEGs. (**C**) Scatter plot of the gene expression correlation between ‘Dan Yang’ and ‘Nuo Mi Lu’ in Group I. (**D**) Scatter plot of the gene expression correlation between ‘Dan Yang’ and ‘Nuo Mi Lu’ in Group II. (**E**) Heat map of the transcript abundance of the shared genes. The columns from left to right are NML-CK, DY-CK, DY-48 h, NML-48 h, NML-12 h, and DY-12 h.

To further compare the differential gene expression in *H. fulva* under heat stress, the gene expression levels of the two varieties in the control group and under heat stress for 12 h and 48 h were selected for study. The results indicate that under heat stress for 12 h and 48 h, a total of 6086 DEGs were identified as shared between the two ‘Dan Yang’ *H. fulva* groups, of which 5059 were upregulated and 1027 were downregulated. Under heat stress for 12 h and 48 h, a total of 6231 DEGs were identified as being shared between the two ‘Nuo Mi Lu’ *H. fulva* groups, with 3108 genes being upregulated and 3123 genes being downregulated. The number of DEGs suggested that the response of *H. fulva* to heat stress was closely related to the duration of heat stress. It could be observed that these shared genes could serve as a broad-spectrum gene response to heat stress in *H. fulva*, while the unique genes exhibit specific expression patterns. A total of 1850 shared DEGs were found across the four control groups, with 1010 being upregulated and 840 being downregulated (Figure 2A,B). This further indicated that these shared DEGs continuously increase with the duration of heat stress treatment. By filtering for genes with an expression fold change of 20, a total of 310 DEGs were obtained, with 104 being upregulated and 206 being downregulated. Visual analysis of the shared DEGs across the four groups was conducted based on the patterns of gene expression levels. The shared DEGs were categorized into two groups based on their expression levels under different conditions. Heat stress suppressed the upregulation of Group I DEGs and induced the upregulation of Group II DEGs (Figure 2E). Genes in Group I and Group II exhibit a co-expression pattern, and there was a positive correlation between the differential expression fold change in ‘Dan Yang’ and ‘Nuo Mi Lu’ genes (Figure 2C,D).

### 3.3. Enrichment of Heat-Tolerance-Related Genes in H. fulva

**Figure 3:**
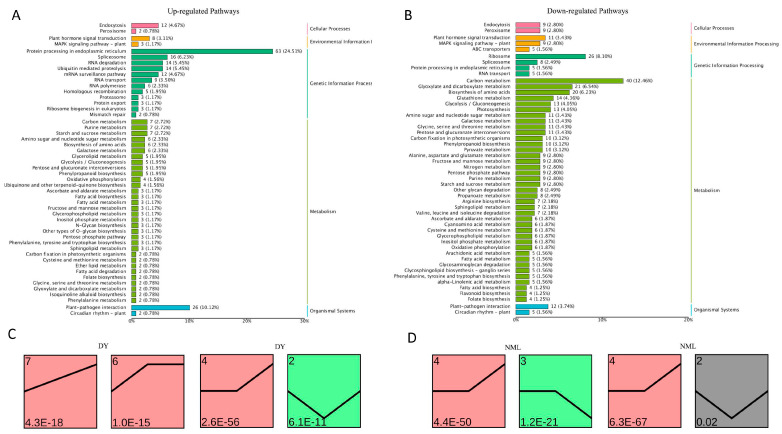
KEGG enrichment of heat-tolerance-related genes in *H. fulva*. (**A**,**B**) bar plots show pathway enrichment presented as number of upregulated and downregulated genes under heat stress vs. control. (**C**,**D**) show the changing trend in genes in the significantly enriched gene profile. The number in the lower left corner represents the significant *p*-value.

To understand the biological relevance of DEGs, the biological pathways involved by the genes of ‘Dan Yang’ and ‘Nuo Mi Lu’ materials were analyzed for enrichment under heat stress and non-heat-stress conditions (Table S2), and a gene expression trend analysis was conducted. In the case of the upregulated genes, the main pathways were as follows: in the endoplasmic reticulum protein processing pathway, there were 63 differentially expressed genes enriched, accounting for 24.51% of the relevant gene set; in the plant–pathogen interaction pathway, 26 differentially expressed genes were enriched, making up 10.12%. Other pathways associated with upregulated genes included the spliceosome, RNA degradation, and ubiquitin-mediated proteolysis pathways (Figure 3A). For downregulated genes, the main pathways included carbon metabolism, ribosome-related processes, amino acid biosynthesis, glutathione metabolism, and photosynthesis. Among them, in the carbon metabolism pathway, 40 differentially expressed genes were enriched, accounting for 12.46%; in the ribosome-related pathway, 26 differentially expressed genes were enriched, accounting for 8.10% (Figure 3B). In the pathways related to plant hormone signal transduction, which involve the abscisic acid, gibberellin, auxin, ethylene, cytokinin, brassinosteroids, jasmonic acid, and salicylic acid pathways, there were 19 differentially expressed genes that were enriched. Significant changes in gene expression related to biological processes and metabolic pathways may be associated with heat stress adaptability or specific biological functions.

Under heat stress, the upregulated and downregulated gene expressions of ‘Dan Yang’ and ‘Nuo Mi Lu’ at three time points (0 h, 12 h, and 48 h) were each divided into seven types of expression trends. In ‘Dan Yang’, upregulated genes were identified as 66 and 35 genes in types 6 and 7, respectively, showing trends in initial continuous upregulation followed by a plateau and continuous upregulation. Downregulated genes were identified as 46 and 143 genes in types 2 and 4, respectively, showing initially decreasing trends followed by an increase and a flat followed by an upward trend (Figure 3C). In ‘Nuo Mi Lu’, upregulated genes were identified as 92 and 161 genes in types 3 and 4, respectively, showing initially plateauing trends followed by a decline and an initial plateau followed by an upward trend. Downregulated genes were identified as 8 and 161 genes in types 2 and 4, respectively, exhibiting initially declining trends followed by an increase and an initial plateau followed by an upward trend (Figure 3D).

### 3.4. WGCNA Analysis Identified Key Genes for Heat Tolerance in H. fulva

#### 3.4.1. Weighted Gene Co-Expression Network Construction and Module Functional Enrichment Analysis

**Figure 4:**
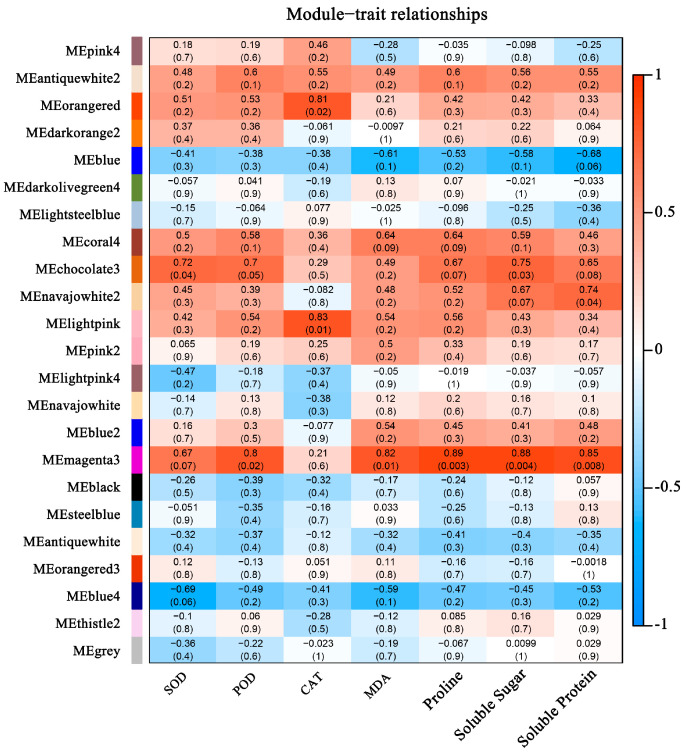
Correlation analysis between modules and phenotypes. SOD stands for Superoxide Dismutase, POD stands for Peroxidase, CAT stands for Catalase, and MDA stands for Malondialdehyde.

By calculating the correlation coefficient of the expression levels among the 24 samples, the samples were well-clustered, and no outlier samples were observed. The dynamic cutting algorithm was used to cluster the genes, and then the module eigenvectors were calculated to optimize the module division, resulting in a total of 23 gene co-expression modules (Figure S2), and the gene co-expression network heatmap was drawn. Based on the correlation analysis between the gene expression modules and heat-tolerant traits, with a Pearson correlation coefficient (r > 0.8) and a significant *p*-value (*p* < 0.03) as screening criteria. The MEorangered, MElightpink, and MEmagenta modules were significantly correlated with physiological and biochemical traits, among which the MEorangered and MElightpink modules were significantly positively correlated with CAT. The gene significance of the MElightpink module was greater than that of the MEorangered module. The MEmagenta module was significantly positively correlated with POD, MDA, proline (Pro), soluble sugar (SS), and soluble protein content (Figure 4).

**Figure 5:**
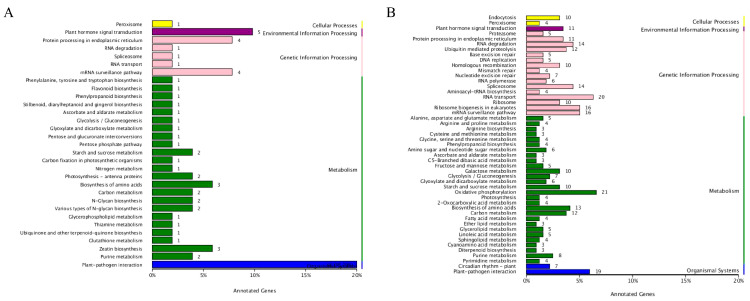
KEGG enrichment analysis. (**A**) MElightpink module analysis. (**B**) MEmagenta3 module analysis.

To reveal the specific functions of each module, a functional enrichment analysis was conducted for the genes in MElightpink and MEmagenta3 modules. The KEGG enrichment analysis of the MElightpink module showed significant enrichment of plant–pathogen interactions, plant hormone signal transduction, protein processing in the endoplasmic reticulum, and the mRNA surveillance pathway (Figure 5A). The MEmagenta3 module was significantly enriched in oxidative phosphorylation, RNA transport, plant–pathogen interactions, etc. (Figure 5B). Plant–pathogen interactions was the most significantly enriched in the two modules. Additionally, pathway enrichment analysis was conducted on the genes in the MEorangered module (Figure S3).

#### 3.4.2. Core Gene Identification

**Figure 6:**
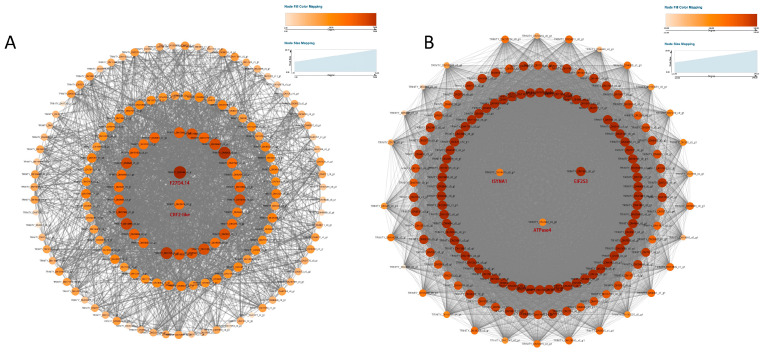
Gene co-expression network of heat-tolerance-related core transcription factors within the MElightpink (**A**) and MEmagenta3 (**B**) modules. The color and the size of the nodes represent the degree value (the degree of correlation among genes). The central point represents the core gene.

Select the top 150 genes with the highest connectivity within the MElightpink and MEmagenta3 modules as the hub genes for each module and predict and annotate the functions of the hub genes. Among them, there were three TFs and DEGs that might have interactions between them that were predicted through WGCNA. The visual analysis was performed using the Cytoscape (v3.9.1) software (Figure 6). In the MElightpink module, there were three transcription factors (one each of bHLH, AP2/ERF, and GRAS) that were associated with the plants’ defense responses. Among them, the number of DEGs related to *CRF2*-like (*TRINITY_DN20076_c0_g1*) was the highest, totaling 47. It was enriched in the plant–pathogen interaction pathway, and the expression levels of this gene, “DY” and “NML”, were both upregulated compared to the control group under heat stress for 12 h, with the highest connectivity among all genes in the MElightpink module being *F27D4.14* (*TRINITY_DN2594_c0_g1*). It was enriched in the plant hormone signal transduction pathway, and its expression levels were both upregulated compared to the control group under heat stress for 12 h in the two varieties. In the MEmagenta3 module, maintaining energy metabolism under high temperature was crucial for plant heat tolerance. *ATPase4* (*TRINITY_DN2442_c0_g2*), *atp1* (*TRINITY_DN4761_c3_g4*), and *cox1* (*TRINITY_DN5736_c0_g3*) were enriched in the oxidative phosphorylation pathway and were differentially upregulated under heat stress in both the “DY” and “NML” varieties. RNA transport helped plants adapt to high temperatures, *EIF2S3* (*TRINITY_DN7895_c0_g1*) was enriched in the RNA transport pathway, had high connectivity, and was differentially upregulated under heat stress in both the “DY” and “NML” varieties. Plant hormones played a crucial role in plant’s response to high-temperature stress. *T2E12.5* (*TRINITY_DN10709_c0_g2*) was enriched in the plant hormone signal transduction pathway and was differentially upregulated under heat stress in both the “DY” and “NML” varieties, with “DY” showing higher expression levels than “NML”. The Inositol metabolism helped plants cope with heat stress and maintain normal physiological functions through osmotic regulation, regulation of key enzymes, and participation in signal transduction pathways. *ISYNA1* (*TRINITY_DN14615_c0_g1*) was enriched in the Inositol phosphate metabolism pathway, had high connectivity, and was significantly upregulated under heat stress in both the “DY” and “NML” varieties, with “DY” showing higher expression levels than “NML”. In the MEorangered module, the MAPK (mitogen-activated protein kinase) signaling pathway played a crucial role in the plant’s response to heat stress. *MMG15.21* (*TRINITY_DN4552_c0_g1*) was enriched in the MAPK signaling pathway plant, had high connectivity, and was differentially upregulated under heat stress in both the “DY” and “NML” varieties, with “DY” showing higher expression levels than “NML”. DNA replication might played a role in plant heat tolerance by maintaining the normal progression of the cell cycle, regulating gene expression, participating in repair mechanisms, and influencing genetic variation. *MCM3* (*TRINITY_DN7055_c0_g3*) was enriched in the DNA replication pathway and was significantly upregulated under heat stress in the “DY” varieties, with “DY” showing higher expression levels than “NML”. Through KEGG enrichment analysis, transcription factor family analysis, and modules associated with heat tolerance in WGCNA, combined with gene annotation, ten candidate genes related to heat stress were screened out, namely *TRINITY_DN20076_c0_g1* (*CRF2-like*), *TRINITY_DN2594_c0_g1* (*F27D4.14*), *TRINITY_DN2442_c0_g2* (*ATPase4*), *TRINITY_DN4761_c3_g4* (*atp1*), *TRINITY_DN5736_c0_g3* (*cox1*), *TRINITY_DN7895_c0_g1* (*EIF2S3*), *TRINITY_DN10709_c0_g2* (*T2E12.5*), *TRINITY_DN14615_c0_g1* (*ISYNA1*), *TRINITY_DN4552_c0_g1* (*MMG15.21*), and *TRINITY_DN7055_c0_g3* (*MCM3*).

**Figure 7:**
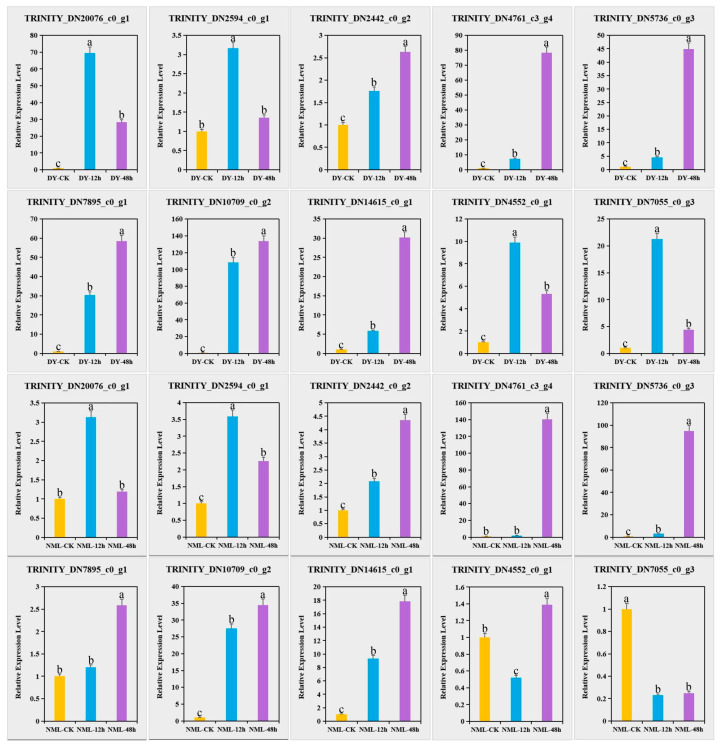
Identification of differentially expressed genes using qRT-PCR. This figure shows the relative expression levels of different genes in various samples. Among them, DY-CK, DY-12 h, and DY-48 h represent the control group, the 12 h treatment group, and the 48 h treatment group of the ‘Dan Yang’ (DY) variety, respectively; NML-CK, NML-12 h, and NML-48 h represent the control group, the 12 h treatment group, and the 48 h treatment group of the ‘Nuo Mi Lu’ (NML) variety, respectively. The ordinate of each bar chart is the relative expression level (Relative Expression Level). Non-identical letters reveal a notable shift between the experimental groups (*p* < 0.05).

Here, the DEGs were selected for qRT-PCR analysis, including plant–pathogen interactions (*TRINITY_DN20076_c0_g1*), plant hormone signal transduction (*TRINITY_DN2594_c0_g1* and *TRINITY_DN10709_c0_g2*), and oxidative transduction phosphorylation (*TRINITY_DN2442_c0_g2*, *TRINITY_DN4761_c3_g4* and *TRINITY_DN5736_c0_g3*), RNA transport (*TRINITY_DN7895_c0_g1*), DNA replication (*TRINITY_DN7055_c0_g3*), Inositol phosphate metabolism (*TRINITY_DN14615_c0_g1*), and the MAPK signaling pathway gene in the plant (*TRINITY_DN4552_c0_g1*) pathway. RT-qPCR was used to analyze the expression changes in these genes in two varieties of *H. fulva* after heat stress. Fluorescence quantitation showed that the relative expression levels of the above genes before and after heat stress were consistent with the transcriptome data, indicating the reliability of the transcriptome data (Figure 7 and Table S3).

## 4. Discussion

High temperature, as a common abiotic stress, can affect the normal growth of plants and limit their geographic distribution [51]. Stress can increase the production of reactive oxygen species and stimulate the activity of antioxidant enzymes such as SOD, POD, and CAT in plants [52]. Under heat stress, high-temperature stress will also destroy the balance of production and the removal of reactive oxygen species in plants, resulting in the production of malondialdehyde and other harmful substances [53]. Plants remove excess ROS by increasing the activity of antioxidant enzymes such as SOD, POD, and CAT, thereby reducing the damage of high temperature to the cell membrane system [54]. The physiological response of plants to heat stress is closely related to species, genotype, and treatment time. This study found that the activity of antioxidant enzymes increased first and then decreased with the time of exposure to high temperatures [55,56]. In this study, SOD, POD, and CAT enzymes in two varieties of *H. fulva* showed a first increasing and then decreasing trend with the increase in heat stress treatment time, which was consistent with the results of other studies. The changing trend in substance content of *H. fulva* was similar to that of other plants in response to heat stress.

Transcriptome techniques have been widely used to study transcriptional regulation in plants under heat stress. Through transcriptome analysis, *Viola × wittrockiana* identified 5708 DEGs that were strongly associated with heat stress, including transcriptional regulators or key components involved in the biosynthesis of HSP, heat shock transcription factors (HSF), and antioxidants [57]. Most plants will identify from hundreds to thousands of DEGs under high-temperature stress, such as *Zea mays L.*, *Populus trichocarpa*, and *Brachystis dipaniculata* [58,59,60]. In our study, 11,773 and 13,212 DEGs were identified in the “DY” variety of *H. fulva* after 12 h and 48 h treatment under heat stress, and 8046 and 9015 DEGs were identified in the “NML” variety after 12 h and 48 h treatment, respectively. In *Rhododendron*, 12,089 and 12,032 DEGs were identified under heat stress at 36 °C and 42 °C [61]. Overall, these results suggest that temperature has a significant effect on gene expression and that plant response to high-temperature stress is achieved through polygenic regulation of plant heat tolerance.

Heat stress can significantly affect a plant’s immune system, thereby altering its defenses against pathogens. Heat stress inhibits specific components of the plant’s immune system, and, under high-temperature conditions, the biosynthetic pathway of salicylic acid (SA) is downregulated, resulting in decreased plant resistance to disease [62]. Research has shown that heat stress not only weakens the immune response of plants, but may also make plants more vulnerable to pathogens, and heat stress may also increase the prevalence of pest and disease transmitters, further threatening plant health [63]. Under heat stress, plant hormones such as jasmonic acid (JA), abscisic acid (ABA), and salicylic acid (SA) play important roles in plant defense signaling pathways. These hormones enhance plant disease resistance by regulating the antioxidant enzyme system, ROS metabolism, and cell wall metabolism [64,65]. However, heat stress may interfere with the normal function of these hormones, thus weakening the plant’s defense response [66]. In this study, CRF2-like (*TRINITY_DN20076_c0_g1*) was enriched in the plant–pathogen interaction pathway. F27D4.14 (*TRINITY_DN2594_c0_g1*) and T2E12.5 (*TRINITY_DN10709_c0_g2*) were enriched in the plant hormone signal transduction pathway, and the gene was significantly upregulated after heat stress at 40 °C. High-temperature stress leads to the accumulation of ROS, which negatively affects plant photosynthesis and cell metabolism. Heat stress increases the production of ROS, which adversely affects plants by damaging macromolecules and cell membranes through oxidation [67]. Under heat stress, plants need to enhance their antioxidant defense systems to protect their oxidative phosphorylation process so as to maintain the normal progress of their energy metabolisms [68]. In this study, *ATPase4* (*TRINITY_DN2442_c0_g2*), *atp1* (*TRINITY_DN4761_c3_g4*), and *cox1* (*TRINITY_DN5736_c0_g3*) genes were significantly increased under heat stress. Both genes are key genes of the oxidative phosphorylation pathway. RNA transport in plant heat tolerance may help plants adapt to high-temperature environments by affecting heat stress gene expression, protein stability, and cell cycle [69]. In addition, under heat stress, plants activate DNA repair mechanisms to prevent damaged cells from continuing to proliferate. In this study, the *EIF2S3* (*TRINITY_DN7895_c0_g1*) gene was enriched in the RNA transport pathway and the *MCM3* (*TRINITY_DN7055_c0_g3*) gene was enriched in the DNA replication pathway. When plants are subjected to high-temperature stress, the MAPK cascade reaction pathway is activated to improve the ability of plants to resist stress. In tomatoes, mutants of the *SlMAPK3* gene showed higher heat tolerance, suggesting that the MAPK signaling pathway plays an important role in regulating plants’ thermal responses [70]. High-temperature treatment also significantly upregulated the expression of certain *MAPK* genes, such as *FvMAPK3* gene in strawberries [71]. Inositol polyphosphokinase (IPMK) plays a key role in plant adaptation to heat stress. In addition, the inositol phosphokinase metabolism pathway crosses with other signal transduction pathways (such as ABA signaling pathway), which further enhances plants’ adaptations to heat stress [72]. In this study, the *ISYNA1* (*TRINITY_DN14615_c0_g1*) gene was enriched in the Inositol phosphate metabolism pathway. The MAPK signaling pathway in plants was enriched with *MMG15.21* (*TRINITY_DN4552_c0_g1*), and this gene was significantly upregulated under heat stress.

## 5. Conclusions

Plant adaptation to high temperature is a coordinated process involving many genes and signaling pathways. In this study, the physiological indexes of *H. fulva* under heat stress were measured, DEGs were screened out based on transcriptome sequencing, and a co-expression network of genes related to heat stress was constructed in combination with WGCNA to explore the mechanisms and genes related to the heat resistance of *H. fulva*. The results show that the activity of SOD, POD, and CAT increased at first and then decreased after reaching different peaks, respectively. The contents of MDA, proline, soluble sugar, and soluble protein increased continuously with the increase in time. According to transcriptome data, 11,773, 8046 and 13,212, 9015 DEGs of “DY” and “NML” were screened under 12 h and 48 h heat stress. Three key gene regulatory modules with high correlations were selected using WGCNA analysis. Ten plant hormone signals, including *TRINITY_DN20076_c0_g1*, *TRINITY_DN2594_c0_g1*, and *TRINITY_DN2442_c0_g2*, were identified in plant–pathogen interaction transduction, oxidative phosphorylation, and other key pathway-enriched genes. This study provides a theoretical reference for the further study of the heat resistance physiology and molecular mechanisms of *H. fulva*.

## Figures and Tables

**Table 1:** Primers used for RT-qPCR.

Gene ID	Forward Primer	Reverse Primer
TRINITY_DN20076_c0_g1	GCCGTCTCCGATGATGATGT	CAGGTCCTCAACGACAGCTT
TRINITY_DN2594_c0_g1	TTTTTGTTGCAGCGACCGAG	TTCGGGGAACTCGTTTCAGG
TRINITY_DN2442_c0_g2	GCCTTGCCAAAGTCTGTGTG	GTCATCGGCTGTGGGAGAAA
TRINITY_DN4761_c3_g4	CCGCTCCGGCACCTATTAAT	TGGCTCAGACCTTGATGCAG
TRINITY_DN5736_c0_g3	ATGGGCCATCTTCAAAGGGG	TTCCGGCAAAGAGACAGGAC
TRINITY_DN7895_c0_g1	TAACAATGCCAGGACGCACT	AGGCACGTTTCCTTTGTGGA
TRINITY_DN10709_c0_g2	TGGTAGCCTTCCCTCTCCTC	CATTTCACCTCCCCCTCCAC
TRINITY_DN14615_c0_g1	ATTGTTCGCACGTGATCCCT	AGACGACGGAACTTGTGCAT
TRINITY_DN4552_c0_g1	AACGTCGTCCTCCATGCATT	ACCACGACGAATTTGACCGA
TRINITY_DN7055_c0_g3	ACGGTCTTGGTCGCTCATTT	CGTGTTGCCATTGTTGGTGT

## Data Availability

The datasets presented in this study can be found in online repositories. The data information is publicly accessible at the National Center for Biotechnology Information (NCBI) under BioProject PRJNA1211532.

## Supplementary Materials

The following supporting information can be downloaded at: https://www.mdpi.com/article/10.3390/plants14050690/s1, Figure S1: Weighted gene co-expression network analysis the determination of soft thresholds; Figure S2: Weighted gene co-expression network analysis cluster analysis of H. fulva response to heat stress; Figure S3: Biological function enrichment analysis of H. fulva in response to heat stress; Table S1: Transcriptome sequencing of *H. fulva* in response to heat stress; Table S2: KEGG enrichment analysis of H. fulva in response to heat stress; Table S3. FPKM values of *H. fulva* in response to heat stress.
