# Supplementary material for: Molecular Mechanisms of Gene Expression Regulation in Response to Heat Stress in Hemerocallis fulva
Source: Plants (Basel). 2025 Feb 24;14(5):690. doi: 10.3390/plants14050690 (PMC11901499; doi:10.3390/plants14050690)
Supplement: Supplementary file 1 [file plants-14-00690-s001.zip › Figure S.pdf]

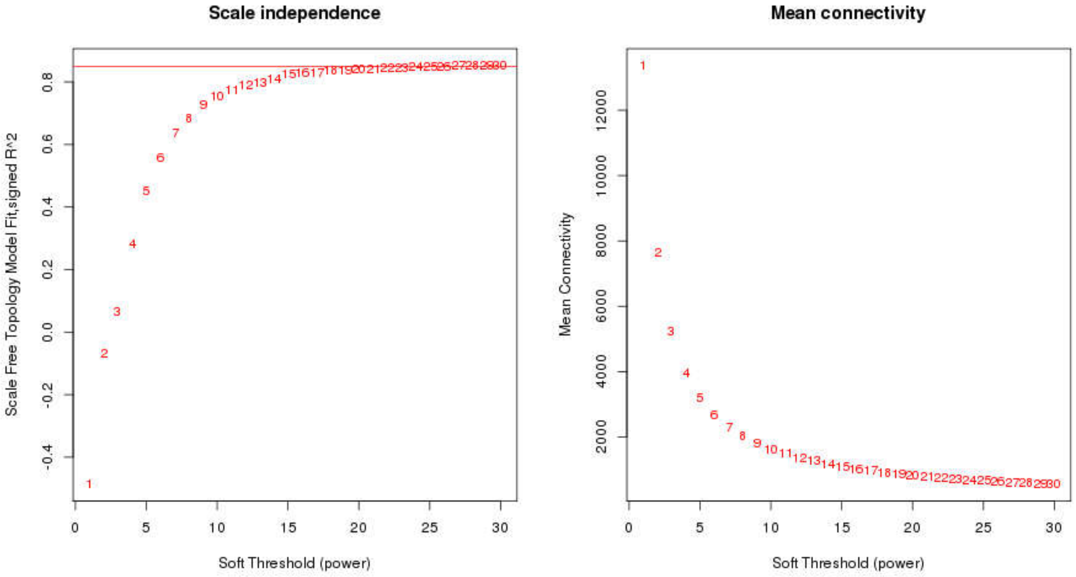

Figure S1. Weighted gene co-expression network analysis the determination of soft thresholds.

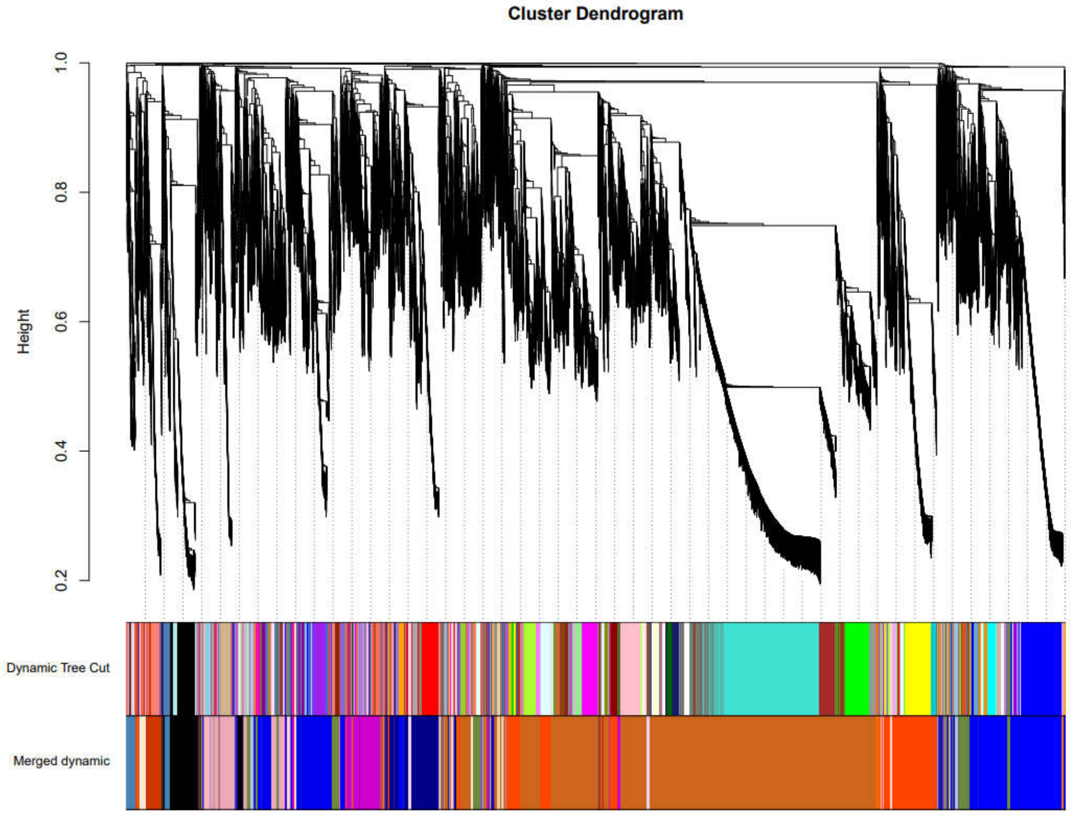

Figure S2. Weighted gene co-expression network analysis cluster analysis of *H. fulva* response to heat stress.

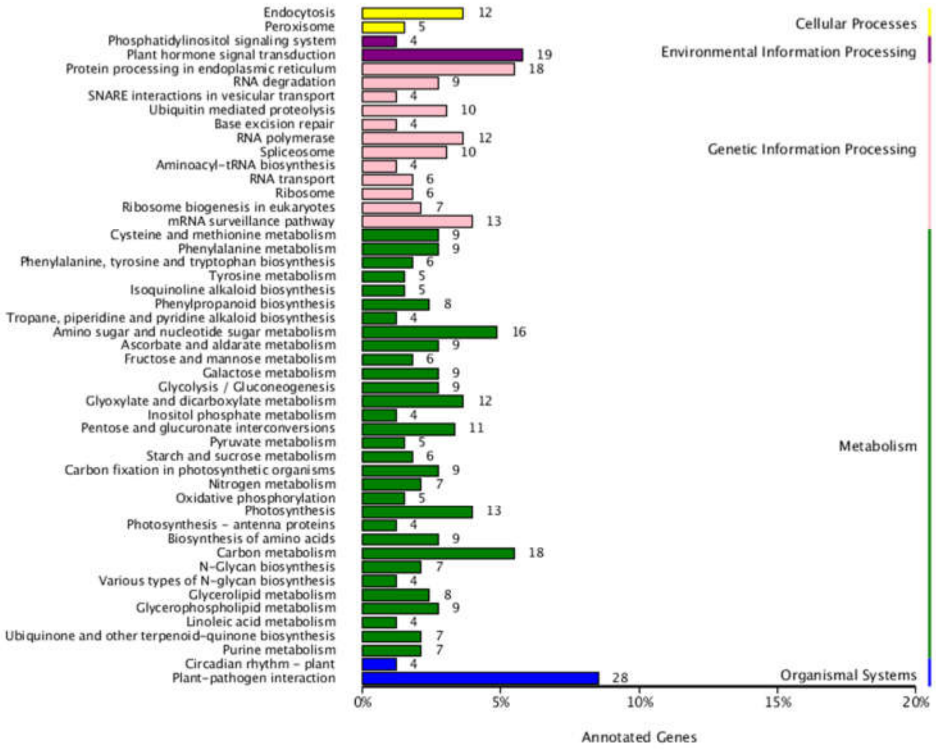

Figure S3. Biological function enrichment analysis of *H. fulva* in response to heat stress.
